# Draft Genome Sequence of *Bacillus* sp. Strain HY001, a High Producer of Isoprene, Isolated from Natto

**DOI:** 10.1128/MRA.01343-19

**Published:** 2020-01-09

**Authors:** Jing Wang, Ming Yan, Cheng Li

**Affiliations:** a College of Biotechnology Engineering, Dalian Polytechnic University, Dalian, China; b College of Biotechnology and Pharmaceutical Engineering, Nanjing Tech University, Nanjing, China; Portland State University

## Abstract

We report the draft genome sequence and annotation of *Bacillus* sp. strain HY001, a high producer of isoprene, isolated from natto. The total 446 contigs have 5,892,114 base pairs containing 6,429 genes and a G+C content of 35.39%.

## ANNOUNCEMENT

Isoprene is the main raw material of synthetic rubber. In addition to plants, many microorganisms can also synthesize isoprene, and it is reported that wild bacteria can produce isoprene only at micromolar levels (1–3). Biosynthesis of isoprene and terpene requires the same isoprene pyrophosphate (IPP) metabolic pathway (4). Natto, a Japanese traditional fermented soybean food, is rich in vitamin K_2_. Vitamin K_2_ is generally believed to be produced by bacteria from the genus *Bacillus* (5). We isolated a new *Bacillus* strain from natto which can produce significantly high levels of isoprene (1.5 mmol/liter, determined by gas chromatography), which may mean that this new strain has a special isoprene synthesis system.

A 5-g natto sample was purchased from a supermarket in Nanjing and was added to 50 ml sterile saline solution. After it was shaken for 1 h, the supernatant was diluted to 10^−8^, and then 0.5 ml of diluent was collected and spread on a casein plate and cultured upside down at 37°C for 36 hours. A single colony of the strain was selected and cultured again on a casein plate at 28°C. A single colony of strain HY001 was selected and cultured in LB broth at 28°C with shaking at 220 rpm for 24 hours. The genomic DNA was extracted using the TIANamp bacterial DNA kit (Tiangen Biotech, Beijing, China) according to the manufacturer’s protocols.

The 16S RNA was sequenced using Genescript (Nanjing, China) after PCR amplification (primers 5′-TTATCGGAGAGTTTGATCCT-3′ [forward] and 5′-TTTCCTCCACTAGGTCGGCG-3′ [backward]). The BLASTn v.2.6.0+ alignment of the 16S rRNA gene sequence to the NCBI nonredundant/nucleotide (nr/nt) database showed 98% sequence identity and 95% query coverage with the Bacillus subtilis strain IAM 12118 16S rRNA gene sequence (GenBank accession number NR_112116). The 16S rRNA gene sequence then was compared to the NCBI’s 16S rRNA database, revealing that the closest related strain was Bacillus toyonensis strain BCT-7112 (GenBank accession number NR_121761; query coverage, 99%; identity, 99.94%).

The library preparation and whole-genome sequencing were carried out by Novogene (Beijing, China). Sequencing libraries were generated using a TruSeq DNA PCR-free sample preparation kit (Illumina, USA) following manufacturer’s recommendations, and index codes were added. The library quality was assessed on a Qubit v.2.0 fluorometer (Thermo Fisher Scientific) and an Agilent Bioanalyzer 2100 system. Finally, the library was sequenced on an Illumina HiSeq 2500 platform, resulting in a total of 934,164 reads.

The read quality was checked using FastQC v.0.11.7 (https://www.bioinformatics.babraham.ac.uk/projects/fastqc/), and the raw paired-end reads were trimmed with Trimmomatic v.0.38, providing approximately 300-fold coverage (6). The assembly of paired-end reads was done with Velvet v.1.2.10 and VelvetOptimiser.pl with default settings (7). After the removal of small contigs of <200 bp, 446 contigs remained. The draft genome sequence of strain HY001 has an average G+C content of 35.39%, an *N*_50_ value of 99,443 bp, and an *L*_50_ value of 17. The 446 contigs have 5,892,114 base pairs.

The draft genome of strain HY001 was analyzed and annotated with the Prokaryotic Genome Annotation Pipeline (PGAP) v.4.8 (8). In total, 6,429 genes were predicted, including 6,297 coding sequences (CDS), 132 RNA genes, and 203 pseudogenes. Using Pathway Tools v.22.0 (9), 270 pathways were identified, including the methylerythritol phosphate pathway II of terpenoid biosynthesis in the genome.

### Data availability.

The draft of the whole-genome shotgun sequence has been deposited in NCBI/EBI/GenBank under BioProject number PRJNA555112, BioSample number SAMN12288858, and GenBank accession number VLTE00000000. The raw sequencing reads have been submitted to the SRA with the accession number SRR10270471.
